# Weight‐Reduction and Safety Profile of Once‐Weekly Non‐Comparable Biotherapeutic Subcutaneous Semaglutide Among People With Obesity: A Real‐World Retrospective Study

**DOI:** 10.1002/hsr2.71745

**Published:** 2026-01-08

**Authors:** Md Rakibul Hasan, Kishore Kumar Shil, Md Shahed‐Morshed

**Affiliations:** ^1^ Department of Endocrinology Medical College for Women and Hospital Dhaka Bangladesh; ^2^ Department of Endocrinology Khulna Medical College and Hospital Khulna Bangladesh; ^3^ Department of Endocrinology Bangladesh Medical University Dhaka Bangladesh

**Keywords:** non‐comparable biotherapeutic semaglutide, Obesity, Semaglutide, Weight loss

## Abstract

**Background and Aims:**

Semaglutide, a once‐weekly injectable glucagon‐like peptide, showed double‐digit weight reduction with an acceptable safety profile in clinical trials and real‐world studies. The efficacy and safety of non‐comparable biotherapeutic (NCB) semaglutide are not adequately reported. Our study aimed to assess the weight reduction and adverse effects of an NCB semaglutide in a real‐world setting.

**Methods:**

This retrospective observational study conducted at three urban private practice settings collected data from an electronic record of 87 people with obesity [age (years): 32.2 ± 11.6, BMI (kg/m^2^): 34.4 ± 4.3, mean ± SD, Female 73(83.9%)] between 7 February 2023 and 31 December 2024 treated with variable doses and durations of NCB semaglutide. The dose, the number of injections administered between visits, weight, side effects, the decision regarding dosing, and patient compliance were recorded at each follow‐up.

**Results:**

The maximum prescribed doses of NCB semaglutide were 0.5 mg per week in 65 (74.7%) of participants, followed by 1.0 mg and 1.7 mg per week in 17 (19.5%) and 5 (5.7%) participants, respectively. Considering available participants of all visits (*n* = 192), 28 (14.6%) participants were lost to follow‐up, NCB semaglutide was ongoing in 32 (16.7%), 23 (12.0%) patients discontinued NCB semaglutide, and one patient died. The mean weight loss was 6.9% and 13.3% at 12 and 24 weeks of follow‐up, respectively. The frequency of reported any side effects and major adverse effects was 59.4% and 9.9%, respectively.

**Conclusion:**

A locally available NCB semaglutide in an uncontrolled, real‐world setting showed acceptable weight loss and safety profiles among people with obesity.

## Summary

- The pure form of semaglutide has shown clinically meaningful weight loss and an acceptable safety profile in clinical trials and real‐world studies.
- Non‐comparable biotherapeutic semaglutide is cheap, but its efficacy and tolerability are not adequately reported.
- This retrospective observational study demonstrated an acceptable weight loss and safety profile of a locally available NCB semaglutide in individuals with obesity in a real‐world setting.

## 1 Introduction

Obesity is a rapidly growing epidemic in the modern era. Globally, 504 million (489–520) women and 374 million (358–391) men are obese, representing an increase of 377 million (360–393) and 307 million (290–324), respectively, from 1990 [1]. A recent meta‐analysis revealed that Bangladesh has a higher prevalence of both obesity (8.9%) and overweight (13.6%) among the young South Asian population [2]. The prevalence of overweight and obesity among women of reproductive age increased significantly from 7.53% and 1.82% in 1999 to 28.37% and 10.77% in 2014, respectively [3]. A nationwide survey from Bangladesh reported that the prevalence of overweight/obesity was 32.6% and 45.6% among males and females, respectively [4]. The increasing prevalence of obesity poses challenges to our healthcare system by increasing the number of obesity‐related morbidities and mortalities. Obesity is associated with an increased risk of various diseases (including Diabetes, Hypertension, Mental and behavioral disease, neurological disease, infection, cancer, musculoskeletal disease, and many others) with varying degrees of association [5, 6]. A minimum of 3−5% weight loss may be sufficient to improve many metabolic complications of obesity [7]. However, more (> 10%−15%) weight loss may improve steatohepatitis. It may be associated with remission of diabetes mellitus (DM) and hypertension [8, 9]. A significant amount of weight loss is possible through lifestyle therapy and oral obesity medications, such as orlistat, phentermine, topiramate, and naltrexone‐bupropion. Several bariatric procedures can achieve more durable weight loss. However, only a few eligible patients avail themselves of this surgery due to its cost, complications, the availability of technical experts, and the requirement for lifelong follow‐up [10]. There is a considerable difference in weight‐reducing efficacy between oral anti‐obesity drugs and bariatric surgery. In recent years, several incretin‐based injectable therapies (liraglutide, semaglutide, tirzepatide, etc.) showed two‐digit number weight loss and are very close to the efficacy of bariatric surgery. The US FDA approved these drugs for the management of obesity. Moreover, they are now approved for the management of obstructive sleep apnea, diabetic kidney disease, heart failure, etc [11, 12].

Semaglutide, a best‐selling weight‐loss drug in the USA in 2024, has been linked to a recent decline in obesity rates [13]. Due to its effectiveness and media attention, high demand has made it unavailable for many patients. This has led to the emergence of compounded and non‐comparable biotherapeutic (NCB) semaglutide products, which are cheaper but not FDA‐approved [14, 15]. Although a compounded medication is made under the supervision of a pharmacist by combining, mixing, or altering the main ingredient to meet a specific patient's need, the compounded semaglutide has raised concerns about impurities, side effects, and unpredictable efficacy [16]. On the other hand, an NCB product is a National Regulatory Agency‐approved copy of an existing biological product that has not been tested for comparability in terms of quality, safety, and efficacy. However, its quality, effectiveness, and safety should be appropriately evaluated following the WHO's guidelines [17].

Asians are more likely to have visceral obesity and may achieve greater benefits from semaglutide compared to other populations [18]. Bangladesh, a developing country in South Asia, is experiencing a surge in non‐communicable diseases, with a particularly alarming rise in obesity rates among both adults and children [19]. However, the high cost of semaglutide in pure form must be paid out‐of‐pocket in Bangladesh, making the pharmaceutical company uninterested in introducing the original brand here [20]. While local brands (NCB products) are available and prescribed for DM and obesity management, their effectiveness and safety have not been thoroughly studied or documented. Our study aimed to retrospectively report the weight‐reducing efficacy, side‐effect profile, and discontinuation rate of locally available NCB semaglutide among Bangladeshi individuals with obesity. We hypothesized that at least 5% mean weight loss over 12 weeks could be achievable with a locally available NCB semaglutide in a real‐world setting.

## 2 Methods

This retrospective observational study utilized electronic records to collect data from three urban private practice settings (Dhaka, Khulna, and Tangail) over 2 years. Participants with obesity who had been prescribed injectable NCB semaglutide were included in the study. Participants who did not complete at least one follow‐up visit and had incomplete data were excluded from the analysis. The first patient was enrolled on February 7, 2023, and data collection continued until December 31, 2024. The Ethical Committee of a Medical College Hospital approved the study protocol on 10th December 2023, Memo No. MCWH/Ethical committee/2023/185.

Using real‐world study data (at least 5% weight loss after 3 months = 70%), with a 95% confidence interval (Z = 1.96), and 10% margin of error (d = 0.1), the sample size was 81 (*n* = Z^2^pq/d^2^). We were able to include 87 participants [21].

Participants were evaluated through a comprehensive history, physical examination, and investigations to diagnose obesity, its complications, and associated comorbidities at the time of diagnosis. We diagnosed obesity using the World Health Organization's criteria, with an Asian−Pacific perspective: in adults, a body mass index (BMI) of ≥ 25 kg/m²; in adolescents, either a BMI percentile of ≥ 95th percentile for age and sex or a BMI of ≥ 35 kg/m². Obesity was further categorized into grade I and grade II based on a BMI cut‐off of 30 kg/m² for adults and ≥ 120% of the 95th percentile for age and sex in adolescents [22]. At baseline and each follow‐up, the investigators counseled participants on diet, physical activity, and behavioral modifications. All the participants were started on 0.25 mg of a locally available NCB semaglutide per week. At each follow‐up, participants' lifestyles, changes in anthropometry, complications, comorbidities, medication adherence, and patient‐reported side effects were assessed and documented on the database and also on the follow‐up form. Then, the decision on drug dose and duration was taken after discussion with participants. From the prescriptions, we documented the baseline characteristics, dose of NCB semaglutide, number of injections administered between two visits, weight, side effects, decision regarding dosing, patient compliance, and other relevant details. We were able to report up to the 5th follow‐up visit of NCB semaglutide therapy. The study flow chart is illustrated in Table 1, showing the number of participants at each follow‐up, along with the decision regarding doses and disposal. We calculated weight changes at each follow‐up after subtracting the baseline weight. Then, we divided it by the baseline weight, followed by multiplication by 100, to obtain percentage weight changes. As there were differences in dose and duration of follow‐ups, we also calculated the cumulative dose of NCB semaglutide in milligrams and duration between follow‐ups in weeks. Additionally, we categorized the percentage of weight loss into the following four categories: < 5%, 5%−9.9%, 10%−14.9%, and ≥ 15% at each visit.

**Table 1:** The study flow chart.

Follow‐up period	Eligible for follow‐up	Available at follow‐up	Decision at follow‐up				Not available at follow‐up			
			↑ dose	↓ dose	↔ dose	Stopped	Total	Lost	Ongoing	Death
1st	87	87	61	0	25	1	0	0	0	0
2nd	86	51	24	1	21	5	35	12	23	0
3rd	46	32	11	1	12	8	14	6	8	0
4th	24	16	2	2	6	6	8	7	1	0
5th	16	6	1	1	1	3	4	3	0	1
Total	259	192	99 (51.6)	5 (2.6)	65 (33.9)	23 (12.0)	61	28	32	1

*Note:* Within parentheses are percentages among the total available cases at all follow‐ups (*n* = 192), ↑ dose increased, ↓ dose decreased, ↔ dose unchanged

We entered, edited, and analyzed our data using SPSS software (version 25.0, IBM Corp., Armonk, NY, USA). We present the qualitative data in terms of frequency and percentage. We checked all the quantitative data using the Shapiro‐Wilk test and found them to be normally distributed; hence, they are expressed as means with standard deviations (SD). To perform a one‐way repeated measures ANOVA among different follow‐up periods for percentage changes in weight, the assumption of sphericity was tested using Mauchley's test. If the assumption was violated, the Greenhouse‐Geisser test was used for interpretation. The frequency of at least 5% weight loss was compared under different baseline characteristics by Pearson's chi‐squared or Fisher's exact test. Any p‐value below 0.05 was considered statistically significant.

## 3 Results

Among the 87 participants, only three continued on NCB semaglutide after the 5th follow‐up. Considering the available participants from all visits (*n* = 192), 28 (14.6%) participants were lost to follow‐up, 32 (16.7%) participants were still on NCB semaglutide, 23 (12.0%) participants discontinued NCB semaglutide, and one patient died (Table 1).

The baseline characteristics of the study participants are shown in Table 2. Among 87 participants, only 10 were adolescents (< 18 years). The majority of the participants were females (83.9%) and had grade II obesity. Nearly 84% of the study participants had at least one co‐morbidity. Dyslipidemia, hypertension, and metabolic dysfunction‐associated steatotic liver disease were the most common comorbidities.

**Table 2:** Baseline characteristics of the study participants, *n* = 87.

Variables	Mean ± SD	Range
Age, years	32.2 ± 11.6	14–64
BMI, kg/m^2^	34.4 ± 4.3	25.4–48.0
Systolic BP, mm‐Hg	123.4 ± 16.7	90–160
Diastolic BP, mm‐Hg	80.4 ± 10.7	60–105

	Frequency	Percent
Sex		
Female	73	83.9
BMI classification *, kg/m^2^		
Grade I obesity, 25–29.9	17	19.5
Grade II obesity, ≥ 30	70	80.5
Number of co‐morbidities		
None	14	16.1
1−3	47	54.0
> 3	26	29.9
Co‐morbidities		
Diabetes mellitus	32	36.8
Hypertension	40	46.0
Dyslipidemia	50	57.5
PCOS	23/73	31.5
MASLD	37	42.5
Hypothyroidism	12	13.8

Abbreviations: BMI, Body mass index; BP, blood pressure; MASLD, Metabolic dysfunction‐associated steatotic liver disease; PCOS, Polycystic ovary syndrome.

**Figure 1:**
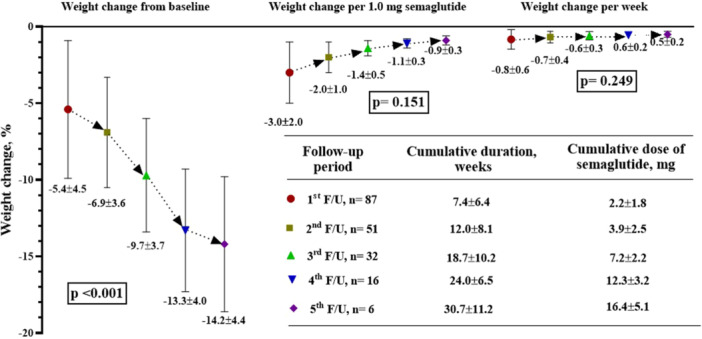
Percentage of weight change at progressive follow‐up in the study population by repeated measures one‐way ANOVA test. (Left portion reporting progressive weight loss patterns in percentage from baseline at progressive follow‐up, right upper portion reporting weight changes per 1.0 mg cumulative dose of NCB Semaglutide and weekly changes of body weight, right lower portion reporting number of patients on subsequent follow‐up, with cumulative duration in weeks, and cumulative dose of NCB Semaglutide).

The percentage of cumulative weight loss increased significantly at each follow‐up period from baseline (F = 20.9, df = 1, *p* < 0.001). Post hoc analysis showed that at the 5th follow‐up visit, the mean weight loss was significantly higher from the 1st [MD = −5.9 (−16.9, −2.3), *p* = 0.015], 2nd [MD = −5.9 (−11.2, −0.6), *p* = 0.031], and 3rd follow‐up [MD = −3.9 (−6.9, −1.0), *p* = 0.014]. The weight loss at the 4th follow‐up visit was also significantly higher than at the 1st follow‐up visit [MD = ‐8.0 (−14.8, −1.3), *p* = 0.023]. The percentage of cumulative weight loss was calculated by dividing the cumulative weight loss by the cumulative dose and duration of NCB semaglutide. Although we observed trends of reduced weight loss with the progression of follow‐up, as measured by both per dose and duration (the mean value at one follow‐up was higher than at the previous visit), there were no significant associations (Figure 1).

**Figure 2:**
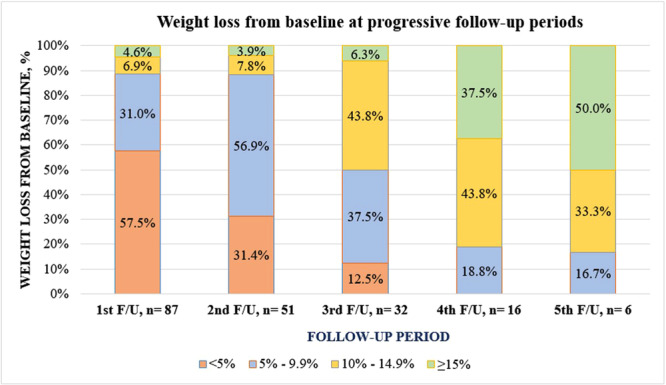
Frequency of participants losing a different degree of body weight (in percentage) at progressive follow‐up (the longer the duration of treatment, the higher the percentages of patients achieving ≥ 5%, ≥ 10%, and ≥ 15% weight loss from the baseline).

Figure 2 illustrates the frequency of participants losing different weight grades at various follow‐up periods. With the progression of time, more people achieved higher grades of weight loss.

The mean duration of the 2nd follow‐up period was 12 weeks. At this time, 35 (68.6%) of the 51 eligible participants had lost at least 5% of their weight. The frequency of at least 5% weight loss was significantly lower in individuals with dyslipidemia (*p* = 0.03) and hypertension (*p* = 0.02) compared to those without these conditions (Table 3).

**Table 3:** Comparison of at least 5% weight loss by different baseline characteristics at 2nd follow‐up, *n* = 51.

Variables	Groups	No. (%)	*p* value
Sex	Female, *n* = 43	30 (69.8%)	0.69‡
	Male, *n* = 8	5 (62.5%)	
Obesity grade	Grade I, *n* = 7	6 (85.7%)	0.41‡
	Grade II, *n* = 44	29 (65.9%)	
Dyslipidemia	Absent, *n* = 21	18 (85.7%)	**0.03** †
	Present, *n* = 30	17 (56.7)	
Hypertension	Absent, *n* = 18	16 (88.9%)	**0.02** †
	Present, *n* = 33	19 (57.6%)	
MASLD	Absent, *n* = 22	16 (72.7%)	0.58†
	Present, *n* = 29	19 (65.5%)	
Diabetes mellitus	Absent, *n* = 29	21 (72.4%)	0.50†
	Present, *n* = 22	14 (63.6%)	
PCOS	Absent, *n* = 40	26 (65.0%)	0.47‡
	Present, *n* = 10	8 (80.0)	

*Note:* Polycystic ovary syndrome (PCOS); Metabolic dysfunction‐associated steatotic liver disease (MASLD).

Within parentheses are percentages over the row total.

^†^ Pearson's chi‐squared test or.

^‡^ Fisher's exact test was done as appropriate.

Side‐effect profiles at different follow‐up periods are shown in Table 4. There was a total of six follow‐up visits. Adding the number of patients in each visit, there were a total of 192 visits. Side effects were frequent in all follow‐ups, especially at later follow‐ups. Nearly 60% of patients reported at least one side‐effect among the total 192 visits. Gastrointestinal side effects were common. The most frequent symptoms were dyspepsia (27.1%), anorexia (24%), nausea (22.4%), diarrhea (20.8%), vomiting (19.3%), etc. Two patients with DM developed mild hypoglycemia. A few patients developed nonspecific abdominal pain, but none had pancreatitis. Among the 23 patients who discontinued NCB semaglutide, 18 did so due to side effects, while others discontinued due to financial issues. One female patient died after completing the 4th follow‐up. The patient developed blisters with multi‐organ failure (according to her husband's description). Her husband gave a history of recent dengue fever within 2 months of her death, which needed hospitalization. However, the exact cause of death was not adequately evaluated.

**Table 4:** Spectrum of patient reported side‐effects of semaglutide at each follow‐up period in the study participants.

Side‐effects	1st F/U, *n* = 87	2nd F/U, *n* = 51	3rd F/U, *n* = 32	4th F/U, *n* = 16	5th F/U, *n* = 6	Total F/U, *n* = 192
Any	47 (54.0)	28 (54.9)	23 (71.9)	12 (75.0)	4 (66.7)	114 (59.4)
Major†	1 (1.1)	5 (9.8)	8 (25.0)	4 (25.0)	1 (1.7)	19 (9.9)
Anorexia	26 (29.9)	7 (13.7)	11 (34.4)	2 (12.5)	0 (0.0)	46 (24.0)
Nausea	12 (13.8)	13 (25.5)	13 (40.6)	4 (25.0)	1 (16.7)	43 (22.4)
Vomiting	17 (19.5)	8 (15.7)	7 (21.9)	5 (31.3)	0 (0.0)	37 (19.3)
Diarrhea	10 (11.5)	14 (27.5)	10 (31.3)	3 (18.8)	3 (50.0)	40 (20.8)
Constipation	3 (3.5)	4 (7.8)	6 (18.8)	1 (6.3)	1 (16.7)	15 (7.8)
Abdominal pain	5 (5.8)	4 (7.8)	6 (18.8)	1 (6.3)	0 (0.0)	16 (8.3)
Dyspepsia/heart burn	21 (24.1)	15 (29.4)	12 (37.5)	4 (25.0)	0 (0.0)	52 (27.1)
Flatulence	2 (2.3)	2 (3.9)	0 (0.0)	0 (0.0)	0 (0.0)	4 (2.1)
Weakness/fatigue	17 (19.5)	2 (3.9)	4 (12.5)	2 (12.5)	1 (16.7)	26 (13.5)
Headache	0 (0.0)	0 (0.0)	1 (3.1)	0 (0.0)	1 (16.7)	2 (1.0)
Belching	0 (0.0)	1 (2.0)	0 (0.0)	0 (0.0)	0 (0.0)	1 (0.5)
Dizziness/vertigo	5 (5.8)	1 (2.0)	2 (6.3)	1 (6.3)	1 (16.7)	10 (5.2)
Hair fall	1 (1.2)	1 (2.0)	6 (18.8)	3 (18.8)	1 (16.7)	12 (6.3)
Feverish feeling/malaise	3 (3.5)	0 (0.0)	0 (0.0)	0 (0.0)	0 (0.0)	3 (1.6)
Injection site pain	3 (3.5)	2 (3.9)	2 (6.3)	0 (0.0)	0 (0.0)	7 (3.7)
Cholelithiasis	0 (0.0)	0 (0.0)	1 (3.1)	0 (0.0)	0 (0.0)	1 (0.5)
Pharyngitis	1 (1.2)	1 (2.0)	0 (0.0)	0 (0.0)	0 (0.0)	2 (1.0)
Hypoglycemia	1 (1.2)	1 (2.0)	0 (0.0)	0 (0.0)	0 (0.0)	2 (1.0)
Pancreatitis	0 (0.0)	0 (0.0)	0 (0.0)	0 (0.0)	0 (0.0)	0 (0.0)
Death	0 (0.0)	0 (0.0)	0 (0.0)	0 (0.0)	1 (16.7)	1 (0.5)
Discontinuation	1 (1.2)	5 (9.8)	8 (15.7)	6 (37.5)	3 (50.0)	23 (12.0)
side effects	1 (1.2)	5 (9.8)	8 (15.7)	4 (25.0)	0 (0.0)	18 (9.4)
Others	0 (0.0)	0 (0.0)	0 (0.0)	2 (12.5)	3 (50.0)	5 (2.6)

*Note:* Within parentheses are percentages over the column total.

^†^ Includes semaglutide discontinuation due to side effects or death.

## 4 Discussion

This retrospective real‐world study showed a significant weight loss with increasing duration of locally available NCB semaglutide treatment. More than two‐thirds of participants who completed a mean of 12 weeks of treatment lost at least 5% of their weight from baseline. Nearly 15% of participants were lost to follow‐up. About 12% discontinued NCB semaglutide, and one patient died.

The pure form of semaglutide, administered at higher doses, outperformed placebo, dulaglutide, and liraglutide in weight reduction among individuals with obesity, regardless of glycemic status [23, 24, 25]. This clinically meaningful weight loss was also observed in the retrospective cohort and real‐world studies [26, 27]. However, we used a locally available brand of NCB semaglutide. We observed the effectiveness of NCB semaglutide for weight reduction in individuals with or without DM, achieving a weight loss of 7% by 12 weeks and 13.5% by 24 weeks. These weight loss outcomes are comparable with both RCTs and real‐world study findings. Even a real‐world study with compounded semaglutide showed a 9.11% weight loss over 4 months, with a mean difference of less than one percent (0.8%) compared to pure semaglutide [28]. Another retrospective study showed nearly 4.7% weight loss over 3 months of compounded semaglutide [29].

In our study, 43.5% of the participants lost at least 5% of their weight from baseline at the 3rd follow‐up period, with a mean duration of nearly 12 weeks. After a mean duration of 24 weeks, 100% of our study participants achieved this goal. Moreover, after a mean duration of 30 weeks, 1 in 2 lost ≥ 15%, and 1 in 3 lost 10%–14.9% of weight from pretreatment weight. Retrospective studies using a pure form of semaglutide showed 54%−70% of participants lost at least 5% of body weight at 3 months and 88% at 6 months [26, 27]. A real‐world study showed that more than 81% of participants lost at least 5% of their weight with 1.0 mg of semaglutide for 12 weeks. Although there were no significant differences in the frequency of 5% weight loss, those who used the pure form of semaglutide achieved 10% and 15% weight loss at higher frequencies than the compounded semaglutide [28]. The initial lower success in our study may be due to the use of lower doses of a locally available NCB semaglutide. However, those who continued for 6 months showed a 100% response. A real‐world study from Greece compared 1.0 mg of semaglutide with 2.0 mg as a maintenance dose after 3 months of 1.0 mg of semaglutide per week. At 6 months, the percentage of weight loss was similar between the groups [27]. However, another retrospective cohort study showed significantly higher weight loss at 3 and 6 months of higher doses (1.7 mg and 2.4 mg) of semaglutide than at lower doses (0.25 mg, 0.5 mg, and 1.0 mg) [26]. Additionally, an RCT showed minimal HbA1C improvement with higher doses (2.4 mg vs. 1.0 mg) of semaglutide despite higher weight loss at the expense of higher adverse effects among people with DM [30]. Therefore, a lower dose of semaglutide, regardless of purity and context, may be beneficial for achieving clinically meaningful weight loss.

In our study, patient follow‐up every 4 weeks for dosage adjustments was not possible due to patient convenience and cost considerations. Additionally, we were unable to increase the dose in nearly 52% of available cases during follow‐ups due to side effects and patients' decisions. A population‐based Danish study found that only 10% could increase the dose every 4 weeks, and nearly 50% continued with a weekly dose of only 1.0 mg due to side effects [31].

Ghusan W, et al. showed a lower weight loss among people with DM than among non‐DM. Weight loss did not significantly vary among different BMI groups [26]. We did not find any significant difference in the frequency of at least 5% weight loss at 12 weeks, regardless of sex, BMI, or DM status. However, those with dyslipidemia and hypertension had a lower frequency of weight loss than those without the conditions. We did not find any studies to compare our findings.

Nearly 60% of study participants developed side effects in our study. The two real‐world studies reported adverse effects of 50% and 65% [26, 27]. These findings are smaller than those of randomized trials due to the use of reduced doses and a shorter duration of semaglutide. In our study, the NCB semaglutide was associated with lower side effects, especially mild ones, than pure semaglutide [28]. Nearly 10% of our study population developed a significant side effect requiring the discontinuation of semaglutide, including one case of fatality. The previously published real‐world studies reported a frequency of nearly 12% and 25% of moderate to severe adverse effects that interfered with or prevented daily normal activities [26, 27]. While RCTs reported pancreatitis and gallbladder disease, real‐world studies did not report any of these [23, 26, 27, 30]. Although we did not observe those side effects, two of our patients developed mild hypoglycemia with concurrent use of sulfonylurea. Besides, one of our patients died. However, we do not have the data regarding the cause of mortality.

About 12% of our study participants discontinued semaglutide, primarily due to adverse effects (9.4%). Other causes included cost and a lack of knowledge. The discontinuation rate was lower in real‐world studies (< 3%) and RCTs (7%) due to adverse effects [23, 26, 27].

Several limitations of this study include its retrospective and observational design, inconsistent follow‐up times, lack of tracking of diet and exercise changes, a short study duration, and a substantial number of lost follow‐ups. Previous studies have demonstrated a low adherence to chronic conditions among Bangladeshi patients due to financial issues, lack of education, stigmatization, and lack of accompanying persons, leading to poor disease status [32, 33].

In conclusion, lower doses of an NCB semaglutide, even with inconsistent follow‐up, may still lead to significant weight loss in real‐world use in people with obesity. Even side effects appear similar to those seen in real‐world studies and randomized controlled trials. However, larger, long‐term studies are needed to validate these results.

## Author Contributions

**Md Rakibul Hasan:** conceptualization, resources, investigation, supervision, project administration, methodology, writing – original draft, writing – review and editing, software, validation, data curation. **Kishore Kumar Shil:** conceptualization, methodology, investigation, writing – review and editing, data curation, writing – original draft. **Md Shahed‐Morshed:** conceptualization, investigation, resources, software, writing – review and editing, visualization, methodology, data curation, writing – original draft, formal analysis.

## Funding

The authors received no specific funding for this work.

## Ethics Statement

The Ethical Committee of Medical College for Women and Hospital, Dhaka, Bangladesh, approved the study protocol (Memo no. MCWH/Ethical committee/2023/185, Date: 10 December 2023).

## Conflicts of Interest

The authors declare no conflicts of interest.

## Declaration

All authors have read and approved the final version of the manuscript. **Md Rakibul Hasan** had full access to all the data in this study and took complete responsibility for the integrity and accuracy of the data analysis.

## Transparency Statement

The lead author M. R. Hasan affirms that this manuscript is an honest, accurate, and transparent account of the study being reported; that no important aspects of the study have been omitted; and that any discrepancies from the study as planned (and, if relevant, registered) have been explained.

## Data Availability

The data that supported the study findings may be available from the corresponding author upon reasonable request.
